# Síndrome do aprisionamento poplíteo e síndrome compartimental crônica dos membros inferiores: desafios no diagnóstico e tratamento

**DOI:** 10.1590/1677-5449.007416

**Published:** 2016

**Authors:** Marcelo José de Almeida

**Affiliations:** 1 Faculdade de Medicina de Marília – FAMEMA, Marília, SP, Brasil.

O atendimento a pacientes jovens, saudáveis e praticantes de atividades esportivas regulares com dores crônicas em membros inferiores constitui um desafio no diagnóstico e no planejamento terapêutico, sendo comum a busca por um cirurgião vascular tanto para identificação como para exclusão de doenças vasculares.

Uma vez descartadas doenças ortopédicas como a síndrome do estresse tibial, fraturas por estresse e tendinopatias, deve-se iniciar uma investigação clínica para identificação da síndrome do aprisionamento da artéria poplítea (SAAP) e da síndrome compartimental crônica de membros inferiores (SCC), causas relativamente frequentes de dores musculares em membros inferiores de jovens, cujo diagnóstico costuma ser tardio. Muitas vezes, é sugerida ao paciente a fisioterapia (em geral, sem bons resultados) ou a interrupção da atividade física, o que limita sua qualidade de vida em termos de lazer, manutenção da saúde ou carreira profissional, como no caso de atletas e militares.

A SAAP é uma doença caracterizada por compressão extrínseca da artéria poplítea e sua incidência é de cerca de 3,4%^1^^,^ 2. Na forma congênita ou clássica, há distúrbios do desenvolvimento embrionário da artéria, veia poplítea ou dos componentes musculotendinosos da fossa poplítea, que resultam em desvios ou compressões arteriais provocadas por essas estruturas anômalas. Na forma funcional, a incidência é desconhecida e não são identificadas malformações anatômicas^3^. Em ambas as situações surgem sintomas de dores no membro acometido, parestesias e, eventualmente, palidez no pé ao se realizar atividades físicas. Pode-se identificar no exame físico uma redução da amplitude dos pulsos no pé durante dorsiflexão ou hiperextensão plantar. O exame de mapeamento dúplex (MD), em geral, permite a visualização da compressão da artéria poplítea durante dorsiflexão plantar, sendo que a ressonância nuclear magnética (RNM) e a angiotomografia computadorizada (ATC) identificam as estruturas envolvidas no encarceramento^4^.

A SAAP funcional surgiu pela falta de encontro de alterações morfológicas no procedimento operatório de casos de SAAP anatômica, apesar do paciente apresentar todos os sinais e sintomas de compressão vascular poplítea^5^. Curiosamente, muitos desses pacientes apresentaram remissão completa dos sintomas após a exploração operatória da artéria poplítea^6^. Esse fato foi atribuído à dissecção e liberação do feixe vascular das estruturas vizinhas pela fasciotomia poplítea.

As avaliações com a RNM em indivíduos com SAAP funcional sintomáticos levaram Turnipseed & Pozniak^7^ a sugerirem que, nesses casos, o encarceramento do feixe neurovascular poplíteo ocorre durante a contração dos músculos gastrocnêmios, os quais empurram o feixe neurovascular poplíteo contra o côndilo femoral. O resultado é a oclusão temporária arterial durante as contrações musculares e o trauma repetitivo do nervo poplíteo. Dessa forma, eles preconizaram para o tratamento da SAAP funcional o acesso medial com abertura do anel solear.

Uma questão interessante é saber se a compressão do feixe poplíteo pode ser identificada tanto em indivíduos sintomáticos como assintomáticos, ou seja, se está presente na população normal. A evolução do MD permitiu que fossem realizadas avaliações em indivíduos assintomáticos^8^. Almeida et al.^9^ avaliaram clinicamente e com MD grupos assintomáticos de indivíduos atletas e não atletas, e identificaram testes positivos em 4,7% dos atletas e 9,5% dos sedentários. Isso demonstra que a compressão arterial poplítea pode ocorrer em todos os indivíduos, independentemente da atividade física. É desconhecido o motivo pelo qual alguns indivíduos com compressão poplítea são sintomáticos e outros não. Melo et al.^10^, ao operarem seis pacientes com SAAP funcional, sugeriram que a atividade física é importante no aparecimento de sintomas e que, nos indivíduos assintomáticos, talvez a atividade física seja insuficiente para o surgimento de queixas clínicas. Esse fato é semelhante à síndrome do desfiladeiro torácico, em que cerca de 30% da população apresenta compressão do feixe neurovascular, e somente pacientes sintomáticos se beneficiam de procedimento operatório. Cumpre ressaltar que, de maneira análoga, a presença de sintomas é importante no diagnóstico da SAAP funcional e, apenas para esses pacientes, pode ser cogitada uma intervenção cirúrgica.

O tratamento do aprisionamento poplíteo geralmente é feito por acesso posterior em baioneta da fossa poplítea e dissecção do feixe vasculonervoso com retirada de faixas e bandas musculares anômalas; no caso da SAAP funcional, é importante a abertura do anel solear, que resulta em alívio da compressão nesse segmento. O traumatismo crônico repetitivo arterial pela SAAP pode levar a uma trombose arterial poplítea, sendo que nesses casos o enxerto interposto com veia safena magna é necessário^11^.

Com relação à SCC, sintomas como dores, câimbras, endurecimento muscular, fraqueza muscular ou formigamento se localizam, em geral, na face anterolateral ou posterior das pernas^12^, irradiando para face lateral do pé e/ou panturrilhas. No exame físico, pode ser palpada alguma tensão na musculatura do compartimento envolvido e pulsos normais. Mais raramente, ocorrem sintomas neurológicos, como formigamento ou parestesias.

As manobras de dorsiflexão e hiperextensão do pé ao MD são normais. Após a realização de exercícios físicos, há endurecimento da musculatura envolvida, com dor à palpação. A medida da pressão do compartimento envolvido ratifica o diagnóstico. Deve-se medir a pressão ao repouso e após exercícios – frequentemente a pressão intracompartimental de 10 a 15 mmHg ao repouso aumenta em 3 a 4 vezes após o exercício e coincide com o surgimento dos sintomas^13^^-^ 16.

Para a SCC, o tratamento tem como objetivo reduzir a pressão intracompartimental através da fasciectomia das lojas acometidas. A fasciectomia, em geral, proporciona melhores resultados que a fasciotomia^17^. A fasciectomia pode ser realizada com uma longa incisão longitudinal na pele ou com variantes em que se realiza uma curta incisão transversal seguida da ressecção longitudinal de uma faixa da fáscia utilizando uma tesoura longa. O tratamento operatório costuma ter bons resultados na remissão dos sintomas^18^.

Por fim, tanto a SAAP quanto a SCC são doenças em que o cirurgião vascular deve estar atento ao diagnóstico preciso e também a um diálogo com o paciente e sua família. O tratamento cirúrgico deverá ser planejado e proporciona melhor qualidade de vida aos pacientes.
