# Supplementary material for: Síndrome do aprisionamento poplíteo e síndrome compartimental crônica dos membros inferiores: desafios no diagnóstico e tratamento
Source: J Vasc Bras. 2016 Oct-Dec;15(4):265–7. [Article in Portuguese] doi: 10.1590/1677-5449.007416 (PMC5829723; doi:10.1590/1677-5449.007416)
Supplement: Translation

# Translation: Popliteal entrapment syndrome and lower limb chronic compartment syndrome: challenges in diagnosis and treatment

**Authors:** Marcelo José de Almeida

**Affiliations:** 1 Faculdade de Medicina de Marília – FAMEMA, Marília, SP, Brazil.

Providing care for young, healthy patients who regularly take part in sport and have chronic lower limb pain is challenging in terms of both diagnosis and planning of treatment and it is common for help to be sought from vascular surgeons, whether to identify or to rule out vascular diseases.

Once orthopedic conditions such as tibial stress syndrome, stress fractures, and tendinopathies have been ruled out, the next step is to initiate clinical investigation to identify popliteal artery entrapment syndrome (PAES) and chronic compartment syndrome (CCS) of the lower limbs. These are relatively frequent causes of muscle pain in young people’s lower limbs although diagnosis is often late, drawn out, and tiring for the patient. Very often the patient is recommended physiotherapy (in general, without good results) or advised to refrain from physical activity, which limits quality-of-life in terms of leisure and maintenance of health and, in the case of athletes or soldiers can be detrimental to careers.

PAES is characterized by extrinsic compression of the popliteal artery. Its incidence is 3.4%.^1^^,^ 2 In the congenital or classical form there are disorders of embryonic development of the artery, the popliteal vein, or the muscles and tendons of the popliteal fossa, causing deviation or compression of the artery, provoked by these anomalous structures. The incidence of the functional form is unknown and it does not exhibit anatomic malformations.^3^ In both forms, the symptoms include pains in the affected limb, paresthesias, and sometimes pallor of the foot during physical activities. Physical examination may detect a reduction in the amplitude of pedal pulses during plantar dorsiflexion or hyperextension. In general, examination with duplex ultrasound (DU) enables visualization of compression of the popliteal artery during plantar dorsiflexion, while magnetic resonance imaging (MRI) and computed tomography angiography will identify the structures involved in entrapment.^4^

The creation of the designation functional PAES was the result of the failure to detect morphological abnormalities during operations on cases of anatomic PAES, despite the patients exhibiting all of the signs and symptoms of popliteal vascular compression.^5^ Interestingly, many of these patients nevertheless exhibited complete remission of symptoms after exploration of the popliteal artery during the operation.^6^ This finding has been attributed to dissection and release of the vascular bundles from the neighboring structures during popliteal fasciotomy.

Investigations with MRI of individuals with symptomatic functional PAES led Turnipseed & Pozniak^7^ to suggest that in these cases entrapment of the popliteal neurovascular bundle occurs during contraction of the gastrocnemius muscles, which push the popliteal neurovascular bundle against the femoral condyle. The result is temporary arterial occlusion during muscle contractions and repetitive trauma to the popliteal nerve. They therefore recommended that functional PAES could be treated via a medial access to release the soleus.

An interesting question is whether compression of the popliteal bundle can be detected in both symptomatic and asymptomatic individuals, i.e., whether it is present in the normal population. The development of DU has made it possible to evaluate asymptomatic people.^8^ Almeida et al.^9^ have studied clinical and DU assessments of groups of asymptomatic people, athletes, and non athletes, finding that tests were positive in 4.7% of the athletes and in 9.5% of sedentary people. This demonstrated that popliteal artery compression can occur in anybody, irrespective of whether they engage in physical activity. However, the reasons why some people with popliteal compression are symptomatic and others are not are not known. Melo et al.^10^ operated on six patients with functional PAES and suggested that physical activity is an important element in emergence of symptoms and that in asymptomatic individuals the level of physical activity is possibly insufficient to provoke symptoms. This would be similar to thoracic outlet syndrome, in that around 30% of the population exhibit compression of the neurovascular bundle, but only symptomatic patients benefit from the operation. It should be emphasized that, in an analogous manner, presence of symptoms is important for diagnosis of functional PAES and surgical intervention should only be considered for these patients.

Treatment of popliteal entrapment is generally accomplished via a posterior bayonet access to the popliteal fossa with dissection of the neurovascular bundle and removal of anomalous bands of muscle; while in the case of functional PAES, it is important to release the soleus, alleviating the compression in this segment. The chronic repetitive arterial trauma caused by PAES can result in popliteal arterial thrombosis and in these cases a bypass graft with a great saphenous vein is needed.^11^

With regard to CCS, symptoms such as pains, cramps, muscle stiffness, muscle weakness, or tingling are generally located at the anterolateral or posterior surfaces of the legs,^12^ irradiating to the lateral surface of the foot and/or calves. Physical examination may reveal palpable tension in the musculature of the compartment involved and normal pulses. More rarely, neurological symptoms such as tingling or paresthesias can occur.

Dorsiflexion and hyperextension of the foot maneuvers are normal on DU. After performing physical exercise, the musculature involved becomes stiff, and is painful on palpation. Measuring the pressure of the compartment involved confirms diagnosis. Pressure should first be measured at rest – often intracompartmental pressures of 10 to 15 mmHg at rest will increase 3 to 4 times after exercise, coinciding with appearance of the symptoms.^13^^-^ 16

Treatment for CCS aims to reduce intracompartmental pressure with fasciectomy of the fascia involved. In general, fasciectomy offers better results than fasciotomy.^17^ The procedure can be performed with a long longitudinal incision of the skin or with variants in which a short transverse incision is followed by a longitudinal fasciectomy with long scissors. The results of surgical treatment are generally good in terms of remission of symptoms.^18^ Finally, both PAES and CCS are diseases in which the vascular surgeon should be alert to the precise diagnosis and should open a dialogue with patients and their families. Surgical treatment should be planned and offers better quality of life for patients.
